# Proteomic Investigation into Betulinic Acid-Induced Apoptosis of Human Cervical Cancer HeLa Cells

**DOI:** 10.1371/journal.pone.0105768

**Published:** 2014-08-22

**Authors:** Tao Xu, Qiuying Pang, Dong Zhou, Aiqin Zhang, Shaman Luo, Yang Wang, Xiufeng Yan

**Affiliations:** Alkali Soil Natural Environmental Science Center, Northeast Forestry University, Key Laboratory of Saline-alkali Vegetation Ecology Restoration in Oil Field, Ministry of Education, Harbin, Heilongjiang, China; Universidad Nacional de La Plata, Argentina

## Abstract

Betulinic acid is a pentacyclic triterpenoid that exhibits anticancer functions in human cancer cells. This study provides evidence that betulinic acid is highly effective against the human cervical cancer cell line HeLa by inducing dose- and time-dependent apoptosis. The apoptotic process was further investigated using a proteomics approach to reveal protein expression changes in HeLa cells following betulinic acid treatment. Proteomic analysis revealed that there were six up- and thirty down-regulated proteins in betulinic acid-induced HeLa cells, and these proteins were then subjected to functional pathway analysis using multiple analysis software. UDP-glucose 6-dehydrogenase, 6-phosphogluconate dehydrogenase decarboxylating, chain A Horf6-a novel human peroxidase enzyme that involved in redox process, was found to be down-regulated during the apoptosis process of the oxidative stress response pathway. Consistent with our results at the protein level, an increase in intracellular reactive oxygen species was observed in betulinic acid-treated cells. The proteins glucose-regulated protein and cargo-selection protein TIP47, which are involved in the endoplasmic reticulum pathway, were up-regulated by betulinic acid treatment. Meanwhile, 14-3-3 family proteins, including 14-3-3β and 14-3-3ε, were down-regulated in response to betulinic acid treatment, which is consistent with the decrease in expression of the target genes *14-3-3β* and *14-3-3ε*. Furthermore, it was found that the antiapoptotic *bcl-2* gene was down-regulated while the proapoptotic *bax* gene was up-regulated after betulinic acid treatment in HeLa cells. These results suggest that betulinic acid induces apoptosis of HeLa cells by triggering both the endoplasmic reticulum pathway and the ROS-mediated mitochondrial pathway.

## Introduction

Betulinic acid (3β-hydroxy-lup-20 (29)-en-28-oic acid) (BA) is a naturally occurring lupane-type triterpene found in the bark of white birch trees. BA plays a crucial role as a source for potential anticancer compounds [1]. In vitro studies have shown that this agent is potently effective against a wide variety of cancer cells, including melanoma, leukemia, colon carcinoma, lung carcinoma, prostate carcinoma and multiple myeloma [2]–[8], at the same time, BA and its analogues could be used as potential therapeutics for HIV-1 infection [9], [10]. It is well known that the mitochondria play an important role in the intrinsic pathway of mammalian apoptosis. A decrease in the mitochondrial inner transmembrane potential is associated with BA treatment, which suggests that the intrinsic mitochondrial pathway is involved in BA-induced apoptosis. The mitochondrial pathway is preceded by the generation of reactive oxygen species (ROS) and is regulated by the Bcl-2 family of proteins, which consists of prosurvival (e.g., Bcl-2, Bcl-XL and Mcl-1) and proapoptotic (Bax, Bad and BH3-only proteins) members [10], [11]. BA has been shown to induce apoptosis in a CD-95 and p53-independent manner by a direct effect on mitochondria [4], [12], [13]. Formations of ROS and protein neosynthesis have been reported to be required for BA-induced cell death [14], [15], [16]. A previous study of apoptosis by BA found that the mitochondrial pathway was inhibited by Bcl-2/Bcl-xL overexpression in human multiple myeloma cells [15], BA induces apoptosis mainly through a mitochondrial pathway with tumor specificity.

Cervical cancer is the third most common type of tumor in women [17]. Several treatments are used for cervical cancer, but each of them has severe adverse effects. Therefore, it is still necessary to find a safer and more efficient treatment. BA is a plant-derived pentacyclic triterpenoid that is toxic to cancer cells, but it has no effect on untransformed cells [1], [2], [8]. It has been reported that BA induces antiproliferative activity on cervical cancer [18], but the molecular mechanisms of this process are not fully understood.

The main purpose of the present study is to investigate the underlying molecular mechanisms of the potential anticancer effects of BA on human cervical cancer cells. Global proteome profiling led to the identification of several pathways that respond to BA treatment and helped to better understand BA's apoptosis-related mechanisms. In this work, we characterized the apoptosis-related protein profile of BA-treated HeLa cells using a comparative 2-DE proteomics approach. The molecular functions and biological processes involved in the mechanism of BA-induced apoptosis will be discussed. Changes in the expression of four genes that encode apoptosis-related proteins were analyzed by performing real-time PCR (qRT-PCR) analysis.

## Materials and Methods

### Cell culture and Proliferation Assay

BA was purchased from Boyle Chemical CO., LTD (Shanghai, China). The human cancer cell line HeLa was purchased from the Tumor Center (Beijing, China). Cells were cultured in RPMI-1640 medium (Hyclone, Logan, UT, USA) with 10% FBS (PAA, Austria), 100 U/mL penicillin and 100 µg/mL streptomycin (Hyclone, Logan, UT, USA). Cells were seeded in 96-well microplates and then incubated at 37°C in 5% CO_2_. After 24 h, the medium was removed and replaced with fresh medium containing various concentrations of BA. Next, 30 µL of 3 mg/mL MTT (Amresco, USA) in PBS was added to each well, and then the plate was further incubated for 4 h. The remaining supernatant was removed, and 150 µL of DMSO was added to each well and mixed thoroughly to dissolve the formazan crystals that formed. After 10 minutes of incubation, the absorbance of each well was read at 490 nm using a BioTek-ELISA plate reader (BioTek Instruments, Winooski, USA). The concentration of BA inhibiting cell growth by 50% (IC50 value) was calculated from dose–response curves. All determinations were performed in triplicate.

### Morphological Changes

To detect morphological changes that occurred during apoptosis, nuclear staining was performed using a 5 µg/mL Hoechst 33258 (Sigma, St. Louis, MO, USA) stain, and samples were visualized using a Nikon fluorescence microscope (ECLIPSE Ti-S, Nikon, Tokyo, Japan) [19].

### Flow cytometry analysis of cell apoptosis

BA-induced apoptosis was observed by AnnexinV-FITC/PI staining (Beyotime Biotech, Beijing, China) according to the manufacturer's instructions. Flow cytometry (Partec Flow cytometry, Germany) was used to analyze differences in apoptosis between control and BA-treated (15 µmol/L, 30 µmol/L and 50 µmol/L) cells at 48 h post treatment. Cells were collected and analyzed by counting normal cells, early-stage apoptotic cells, and late-stage apoptotic/necrotic cells in three fields of view of the microscope. The data acquisition and analysis were performed using FloMax software. Cells were processed as described in the manufacturer's protocol and analyzed in triplicate.

### Protein preparation for 2-DE

Hela cells treated with 30 µmol/L BA were harvested after 48 h incubation. Cells were washed twice with ice-cold PBS, then extracted with lysis buffer containing 7 mol/L urea, 2 mol/L thiourea, 4% CHAPS, 2% pH 3-10/4-7 linear IPG buffer (GE Healthcare, USA), 20 mmol/L dithiothreilol (DTT, Sigma, USA), 40 mmol/L Tris (Sigma, USA) and complete mini EDTA-free protease inhibitor cocktail (Roche, USA). After sonicating10 times for 30 s with 30 s pauses in an ice-water bath and then incubating at 4°C for 1 h, cell lysates were centrifuged at 14,000 rpm for 1 h at 4°C. Proteins in the resulting supernatant were quantified according to Bio-Rad protein assay reagent according to the Bradford method (Bio-Rad, Hercules, USA) [19].

### Two-dimensional gel electrophoresis

Cell protein lysates (130 µg) were mixed with rehydration solution containing 7 mol/L urea, 2 mol/L thiourea, 2% CHAPS, 0.5% IPG buffer and 0.002% bromophenol blue and DTT to a final volume of 450 µL. Precast 24-cm immobilized pH gradient strips (IPG, pH 3–10, pH 4–7, GE Healthcare) were rehydrated for 12 h at 30 V. The separation on an Ettan IPGphor 3 (GE Healthcare, Germany) was performed with the following parameters: 100 V for 2 h, 200 V for 1 h, 500 V for 1 h 1000 V for 1 h and 8000 V for 3 h, followed by a step-and-hold pattern of 8000 V for 8 h. After isoelectric focusing, the strips were incubated for 15 min in equilibration solution I (6 mol/L urea, 2%SDS, 30% glycerol, 1% DTT, 0.002% bromophenol blue, 50 mmol/L Tris-HCl, pH 8.8) and for another 15 min in equilibration solution II (1% DTT was replaced with 2.5% iodoacetamide). Subsequently, IPG strips were moved to the top of polyacrylamide gels and embedded using 0.5% (w/v) agarose solution containing bromophenol blue. Two-dimensional SDS–PAGE was carried out at 25°C and 3.5 w/gel for 30 min and then 17.5 w/gel for 4 h 30 min until the bromophenol blue dye front arrived at the bottom of the gels using the Ettan DALT six system (GE Healthcare). Gels were fixed in a mixture of 40% ethanol and 10% glacial acetic acid overnight. Proteins were visualized by silver staining, and gel images were acquired using an ImageScanner (GE Healthcare) with Image Master 2D Platinum Software Version 7.0 (GE Healthcare). In order to obtain reliable results from 2-DE images, spots were well resolved in the three biological replicates. A measurement was carried out for each biological replicate, and normalized volumes were computed using the total spot volume normalization procedure of the software. The normalized volume of each spot was assumed to represent its expression abundance. Only those spots that changed consistently and significantly (more than 1.5-fold and *p*<0.05) were selected for mass spectrometry analysis.

### Mass spectrometry and protein classification

Peptide MS and MS/MS were performed on an ABI 5800 MALDI-TOF/TOF Plus mass spectrometer (Applied Biosystems, USA). Data were acquired in a positive MS reflector using a CalMix5 standard to calibrate the instrument (ABI5800 Calibration Mixture). Both the MS and MS/MS data were integrated and processed by using the GPS Explorer V3.6 software (Applied Biosystems, USA) with default parameters. Based on combined MS and MS/MS spectra, proteins were successfully identified based on 95% or higher confidence interval of their scores in the MASCOT V2.3search engine (Matrix Science Ltd., U.K.), using the following search parameters: NCBInr-humandatabase; trypsin as the digestion enzyme; one missed cleavage site; fixed modifications of Carbamidomethyl (C); partial modifications of Acetyl (Protein N-term), Deamidated (NQ),Dioxidation (W), Oxidation(M); 100 ppm for precursor ion tolerance and 0.5 Da for fragment ion tolerance.

Based on the PANTHER classification (version 7.2, http://www.pantherdb.org/), molecular function and biological process of the corresponding identified proteins were classified [20]. The protein interaction network generated with STRING (version 9.05, http://string-db.org) revealed the functional links between different proteins [21], [22].

### Measurement of oxidative stress

The levels of intracellular ROS were determined using a ROS assay kit (Beyotime Biotech, China) following the manufacturer's protocol. Cells were harvested after 48 h of 30 µmol/L BA treatment and then washed twice with PBS and incubated with DCFH-DA (10 µmol/L) at 37°C for 40 min in a darkroom for final analysis by flow cytometry. All determinations were performed in triplicate.

### RNA isolation and qRT-PCR analysis

Total RNA was isolated using Trizol reagent (Invitrogen, USA). Reverse transcription was performed using a PrimeScriptTM reverse transcriptase (Takara, Tokyo, Japan), and transcripts were then subjected to qRT-PCR using a SYBR Green PCR Reagents Kit (Takara, Tokyo, Japan). Quantitative assays were performed in triplicate using 1 µL of cDNA (1∶10 dilution) and a SYBR Green Master mix (Takara, Tokyo, Japan) and analyzed using an ABI 7500 sequence detection system (Applied Biosystems, USA). The amplification of glyceraldehyde-3-phosphate dehydrogenase (GAPDH) was used as an internal control and to normalize the data. The details of the used primers are shown in Table 1 [23].

**Table 1:** Oligonucleotides and expected sizes of PCR products for qRT-PCR analysis of differentiation marker genes.

	Primer sequence (5′ -> 3′)	Product size(bp)	Anneal Temperature (°C)
*bax*	AGAAGGCACTAATCAAGTCAAGGTC	438	61
	TTTCTGACGGCAACTTCAACTGGGG		
*bcl-2*	AAGGGCATTTTTCCCATCGCTGTCC	350	61
	TCTGTGCCTGTAAACATAGATTCGC		
*14-3-3β*	GTTTGTTGTCTCCAGATGCCACTTC	146	61
	GAAGAAGCAGCAGATGGGCAAAGAG		
*14-3-3ε*	TTCTTTATTCTGCTCTTCACCGTCA	253	61
	AGAACTTCCACCAACGCATCCTATT		
*GAPDH*	TTGGTTGAGCACAGGGTACTTTATT	166	61
	CACCAGGTGGTCTCCTCTGACTTCA		

### Statistical analysis

The data are presented as the mean ± standard deviation (SD) of triplicate samples. Statistical analysis was performed with statistics software (SPSS version 17.0). Data were analyzed using one-way analysis of variance (ANOVA) followed by Bonferroni's multiple comparisons. A value of *p*<0.05 was considered statistically significant.

## Results

### Effect of BA on proliferation and apoptosis of HeLa cells

**Figure 1:**
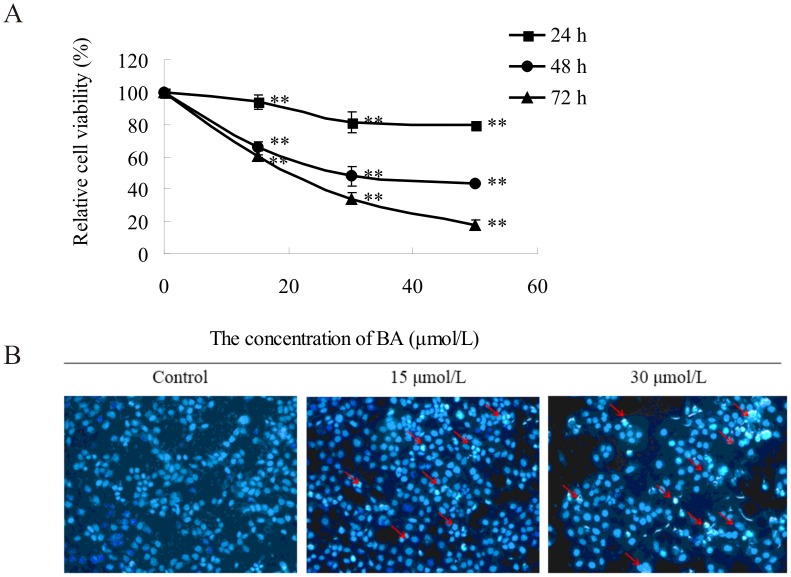
Effects of BA on the proliferation and apoptosis of HeLa cells. (A) Effect of BA towards Hela cells as determined by the MTT assay. Cells were treated with concentrations of BA (0 µmol/L, 15 µmol/L, 30 µmol/L, 50 µmol/L) for indicated time (24 h, 48 h, 72 h). The values for each BA concentration tested represent mean of three experiments, datas are presented as mean average ± SD; **p<0.01 compared with the control group (0 µmol/L BA). (B) Hoechst 33258-staining of HeLa cells treated with 15 µmol/L, 30 µmol/L BA. Red arrows indicate several apoptotic cells with typical condensation of chromatin.

HeLa cells were treated with different concentration (15 µmol/L, 30 µmol/L, 50 µmol/L) of BA for 24 h, 48 h, and 72 h. The cell viability displayed a general decline with increasing concentrations of BA in the medium and treatment duration as analyzed by an MTT assay (Fig. 1A). This result suggests that BA inhibits the proliferation of HeLa cells in a dose- and time-dependent manner. The IC_50_ was 30.42±2.39 µM when HeLa cells were treated with BA for 48 h.

To determine whether the antiproliferative activity induced by BA on HeLa cells was mediated by the induction of apoptosis, we evaluated apoptosis by morphological examination using fluorescence microscopy and flow cytometry. After treatment with 15 µmol/L and 30 µmol/L BA for 48 h, HeLa cells were stained with 5 µg/ml Hoechst 33258 and visualized using fluorescent microscopy. The typical morphology of apoptosis was detected in BA-treated HeLa cells (Fig. 1B). This revealed there was a significant increase in the percentage of apoptotic cells after BA treatment compared to the control cells. In addition, BA caused dose-dependent apoptosis as indicated by annexinV-FITC/PI using flow cytometry. The total percentage of apoptotic cells was 20.53±0.81% (13.45±0.80% of cells early apoptotic and 7.07±0.51% of cells late apoptotic) and 38.56±1.79% (30.92±1.22% of cells early apoptotic and 7.64±0.53% of cells late apoptotic) for cells treated with 30 µmol/L and 50 µmol/L of BA at 48 h, respectively (as shown in Fig. S1).

### 2-DE analysis of differences in protein expression induced by BA

**Figure 2:**
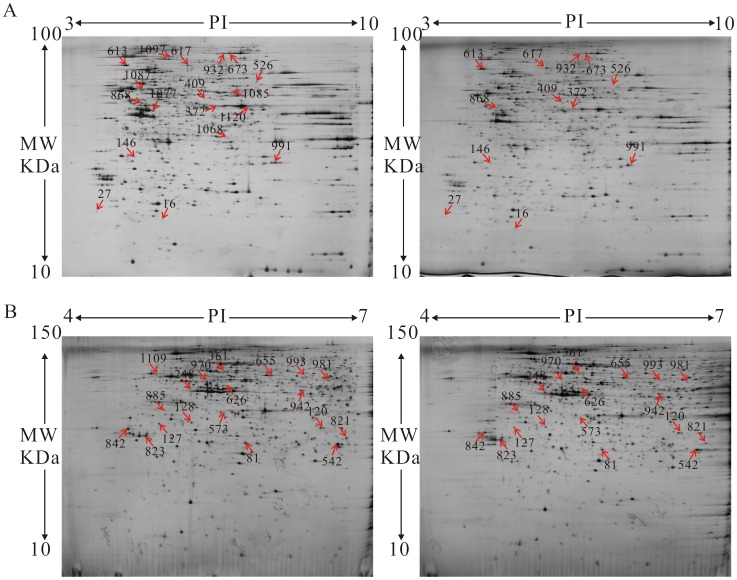
Proteomic analysis of BA-induced Hela cells was conducted by two-dimensional gel electrophoresis (2-DE). (A) 2-DE was performed with 130 µg protein using 24-cm pH 3–10 NL IPG strips and 12.5% SDS–PAGE. (B) 2-DE was performed with 130 µg protein using 24-cm pH 4–7 NL IPG strips and 15% SDS–PAGE. The gels were visualized by silver staining and analyzed with Image Master Software.

Proteomics is a powerful platform for investigating protein expression and modification in response to specific physiological conditions in biological systems. The proteomes of control and 30 µmol/L BA-treated HeLa cells were analyzed by 2-DE. Two gradient-range IPG strips (pH 3–10 and 4–7) were used as the first dimension. Representative 2-DE protein patterns in the pI region of 3–10 showed that proteins mainly focused on the pH range from 4.2 to 7.0 (Fig. 2A). To further separate this sample, narrow-range pH gradients (4–7) were used (Fig. 2B), enabling us to increase both the protein load on the gels and the resolution of the proteins in that range. The protein spots with alterations over 1.5-fold (*p*<0.05) were then subjected to protein identification by MALDI TOF/TOF-MS analysis (Table S1). A total of 18 protein spots from the wide range IPG-based 2D gels (pH 3–10) and 19 protein spots from narrower range IPG-based 2D gels (pH 4–7) showed reproducible and statistically significant changes in BA-treated samples compared to control samples (Fig. 2). Proteins from all the protein spots were successfully identified by MS and MASCOT database searches with high confidence (Table 2). Protein profiling (pH 3–10) revealed 5 up-regulated proteins and 13 down-regulated proteins in BA-treated cells. In addition, protein profiling (pH 4–7) revealed 1 up-regulated protein and 18 down-regulated proteins in BA-treated cells. Unexpectedly, there was only 1 protein (gi4826760) in the overlap between the pH 3–10 (Spot NO. 868) and pH 4–7 (Spot NO. 626) gels.

**Table 2:** Proteins identified for which the level of expression changed significantly (*p*<0.05) after the treatment of 30 µmol/L BA for 48 h.

Spots NO.^a^	Protein Name^b^	Abbreviate name^c^	Accession NO.^d^	MW (kDa)/pI^e^	Protein Score^f^	Regulation^g^
16	phosphomevalonate kinase	PMVK	gi|5729980	5.56/22152	466	↑
27	interferon-gamma inducing factor precursor	IL18	gi|1899242	4.54/22466	88	↑
146	glyoxalase domain-containing protein 4	GLOD4	gi|4929769	8.99/55832	136	↑
613	78 kDa glucose-regulated protein precursor	HSPA5	gi|386758	5.03/72185	1064	↑
617	lamin A protein, partial	LMNA	gi|386856	6.4/57686	151	↑
248	cargo selection protein TIP47	PLIN3	gi|3095186	5.00/47254	53	↑
372	alpha-enolase isoform 1	ENO1	gi|4503571	7.01/47481	140	↓
409	ruvB-like 1	RUVBL1	gi|4506753	6.02/50538	89	↓
526	UDP-glucose 6-dehydrogenase isoform 1	UGDH	gi|4507813	6.73/55674	279	↓
673	elongation factor 2	EEF2	gi|4503483	6.41/96246	722	↓
868	heterogeneous nuclear ribonucleoprotein F	HNRNPF	gi|4826760	5.38/45985	88	↓
932	elongation factor 2 isoform X1	EEF2	gi|530425157	5.77/63494	234	↓
991	guanine nucleotide-binding protein subunit beta-2-like 1	GNB2L1	gi|5174447	7.6/35511	167	↓
1068	TIMM50 protein	TIMM50	gi|14290586	8.56/39014	280	↓
1077	guanine deaminase isoform b	GDA	gi|4758426	5.44/51484	345	↓
1085	adenylyl cyclase-associated protein 1	CAP1	gi|5453595	8.07/51926	67	↓
1087	alpha-tubulin	TUBA1A	gi|37492	5.02/50810	68	↓
1097	motor protein	IMMT	gi|516764	5.71/79830	252	↓
1120	6-phosphogluconate dehydrogenase, decarboxylating	PGD	gi|40068518	6.8/53619	217	↓
81	heat shock protein 27	HSPB1	gi|662841	5.56/27458	345	↓
120	endoplasmic reticulum resident protein 29 isoform 1 precursor	ERP29	gi|5803013	6.35/32445	93	↓
127	nudix hydrolase NUDT5	NUDT5	gi|6694937	4.76/33277	49	↓
128	microtubule-associated protein RP/EB family member 1	MAPRE1	gi|6912494	5.00/33773	407	↓
361	alpha-internexin	INA	gi|14249342	5.33/57148	84	↓
542	chain A, Horf6 A novel human peroxidase enzyme	PRDX6	gi|3318841	6.5/27400	566	↓
573	BTB/POZ domain-containing protein KCTD12	KCTD12	gi|19923973	5.34/37615	52	↓
626	heterogeneous nuclear ribonucleoprotein F	HNRNPF	gi|4826760	5.36/49502	117	↓
655	chain A, tapasin ERp57 heterodimer	PDIA3	gi|220702506	5.82/55312	336	↓
821	phosphoglycerate mutase 1	PGAM1	gi|4505753	6.59/29254	291	↓
823	14-3-3 protein beta	YWHAB	gi|4507949	4.67/29378	273	↓
842	14-3-3 protein epsilon	YWHAE	gi|5803225	4.53/31499	322	↓
885	elongation factor 1-delta isoform 2	EEF1D	gi|25453472	4.8/38175	152	↓
942	dual specificity mitogen-activated protein kinase kinase 2	MAP2K2	gi|13489054	6.15/47554	73	↓
970	mutant beta-actin (beta'-actin)	ACTB	gi|28336	5.16/51529	67	↓
981	T-complex protein 1, Beta subunit (TCP-1-BETA)	CCT1	gi|1871210	6.4/52518	45	↓
993	selenium-binding protein 1 isoform 1	SELENBP1	gi|16306550	4.15/53639	73	↓
1109	unnamed protein product	P4HB	gi|35655	4.75/55600	230	↓

a Assigned spot number as indicated in Fig. 2.

b Each protein spot was identified based on mass spectra of tryptic peptides obtained by MALDI-TOF mass spectrometry.

c Abbreviate name is connected with signal network.

d Accession numbers of the identified protein spots were obtained from the NCBInr-human data base.

e Theoretical molecular mass (Da) and pI.

f Mascot score reported after searching against the NCBInr database.

g The protein spots altered over 1.5-folds.

### Functional categories of differentially expressed proteins

**Figure 3:**
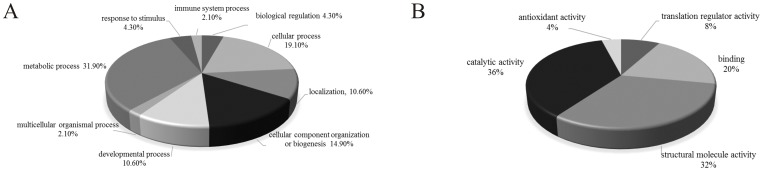
Categorization of BA-regulated proteins and the validation of differentially expressed proteins. Based on the PANTHER classification and gene category, biological process (A) and molecular function (B) of the corresponding identified proteins were classified.

To better classify the identified proteins, two independent categories of gene ontologies were used to describe the function of the gene products: molecular function and biological processes, as identified by the PANTHER classification system. Because EEF (gi530425157, spot 932) was determined to be the same as EEF2 (gi4503483, spot 673) and GLOD4 (gi4929769, spot 146) was not identified by the PANTHER classification system, 34 changed proteins were analyzed by the software. The identified proteins were categorized into 9 groups based on biological processes: metabolic processes (31.90%), cellular processes (19.10%), cellular component organization or biogenesis (14.90%), developmental processes (10.60%), localization (10.60%), biological regulation (4.30%), response to stimulus (4.30%), multicellular organismal processes (2.10%) and immune system processes (2.10%) (Fig. 3A). BA treatment inhibited protein folding (spot 81, 655, 1109, 613, 981), which is important for protein biosynthesis. In addition, glycolysis (spot 372), translation (spot 673, 885) and mRNA splicing (spot 868) are involved in metabolic processes and were down-regulated by BA-treatment. BA suppresses most biological processes to block normal metabolic processes, such as cellular processes (spot 361, 128, 1087, 970, 823, 991, 842), developmental processes (spot 361, 128, 1087, 970), localization (spot 120, 970, 991), responses to stimulus (spot 81), multicellular organismal processes (spot 81), and immune system processes (spot 81). In fact, some proteins are involved in multiple biological processes, and proteins that were involved in cellular processes also contributed to cellular component organization (spot 361, 128, 1087, 970). According to their molecular function, the modulated proteins were classified into 5 groups: catalytic activity (36.00%), structural molecule activity (32.00%), binding (20.00%), translation regulator activity (8.00%), and antioxidant activity (4.00%) (Fig. 3B). In the catalytic activity category, protein disulfide isomerase activity (spot 655, 1109) and oxidoreductase activity (spot 542, 526, 1120, which are in the mitochondrial electron transport chain) were inhibited by BA treatment. Simultaneously, antioxidant activity (spot 542) was also decreased by BA treatment. In addition, most proteins involved in structural molecule activity (spot 81, 361, 1085, 1087, 970), binding (spot 128, 573, 868) and translation regulator activity (spot 673, 885) were reduced significantly in BA treated HeLa cells.

**Figure 4:**
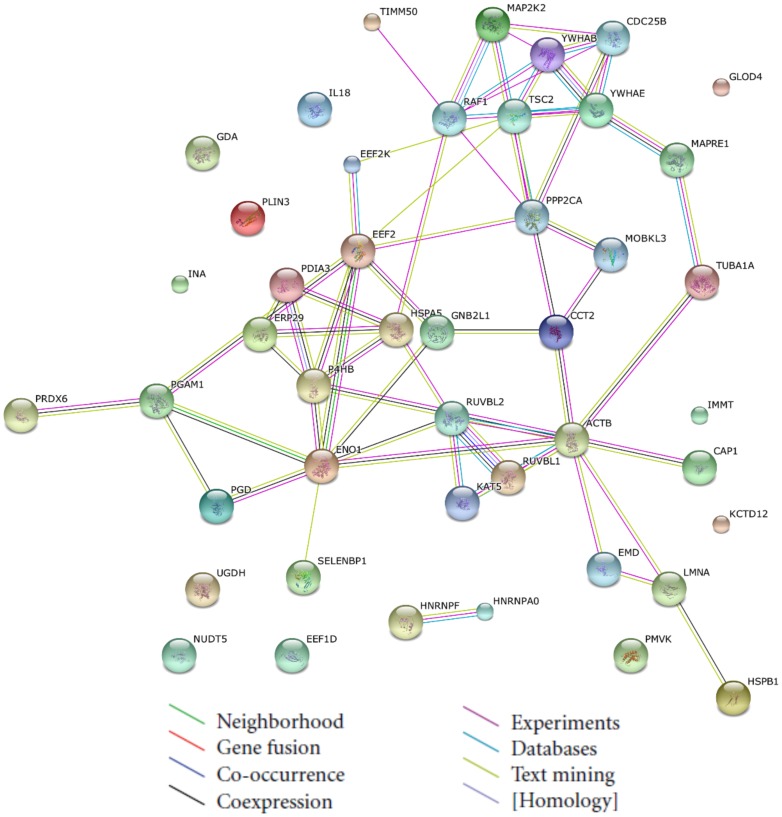
Construction of integrated signaling networks. The changed proteins from proteomic analysis were uploaded to the STRING tool to identify functional signaling networks. The predicted functional links consist of up to eight lines: one color for each type of evidence.

A protein interaction network was generated by STRING, which offers an improvement in functional analysis (Fig. 4). The protein interaction network provides a platform for analyzing the identified proteins' functions, protein–protein interactions, biological pathways and molecular functions. Consequently, four major networks were generated: (1) neurotrophin signaling pathway, (2) long-term depression, (3) arrhythmogenic right ventricular cardiomyopathy, and (4) VEGF signaling pathway. MAP2K2 (spot 942), YWHAB (spot 823) and YWHAE (spot 842) were the central proteins of the interacting networks, and their levels were decreased by BA treatment, which may play an important role in the neurotrophin signaling pathway. Of the identified proteins, the 14-3-3 proteins are involved in many physiological pathways, and these proteins have been shown to be responsible for the growth and apoptotic capacity of many malignant tumors.

### ROS generation in BA-treated HeLa cells

**Figure 5:**
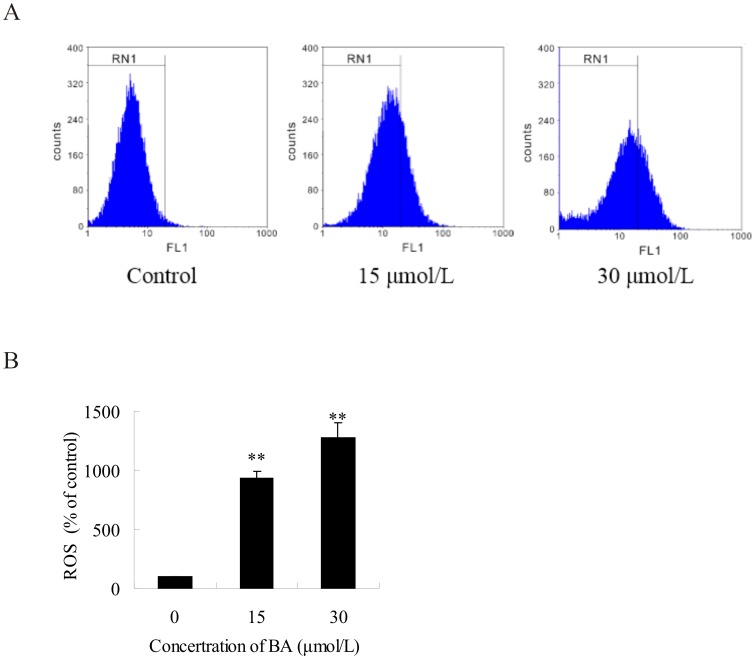
Flow cytometric analysis of reactive oxygen species (ROS) in BA-treated cells. HeLa cells were treated with 15 µmol/L, 30 µmol/L BA for 48 h and then incubated with 10 mmol/L DCFH-DA for 40 min. The fluorescent intensity of DCFH was measured by flow cytometry. (A) Actual spectra from a representative single experiment. (B) The fluorescence intensity of stained cells was determined by flow cytometry. Columns show mean values of three experiments (±SD). **p<0.01 compared with the control group (0 µmol/L BA).

ROS play an important role in apoptosis induction because it is involved in mitochondrial membrane permeabilization and cell death induction. According to the protein profiling of the BA-treated cells, UDP-glucose 6-dehydrogenase (spot 526), 6-phosphogluconate dehydrogenase (spot 1120) and peroxidase enzyme (spot 542) were involved in the biological process of oxidase stress response, which correlates with BA-induced apoptosis of the HeLa cells. The level of ROS production was detected after treatment of the HeLa cells with BA (15 µmol/L, 30 µmol/L) for 48 h. The level of ROS in BA-treated cells increased significantly. The level of ROS in BA-treated cells was 9.28-fold and 12.77-fold higher than the level of ROS in control cells for treatments of 15 µmol/L and 30 µmol/L, respectively, and an up-regulated tendency was observed throughout the experiment (Fig. 5). These results suggest that BA induces apoptosis through the activation of ROS-mediated cell-death mechanisms in HeLa cells.

### qRT-PCR analysis of genes involved in BA-induced cell apoptosis

**Figure 6:**
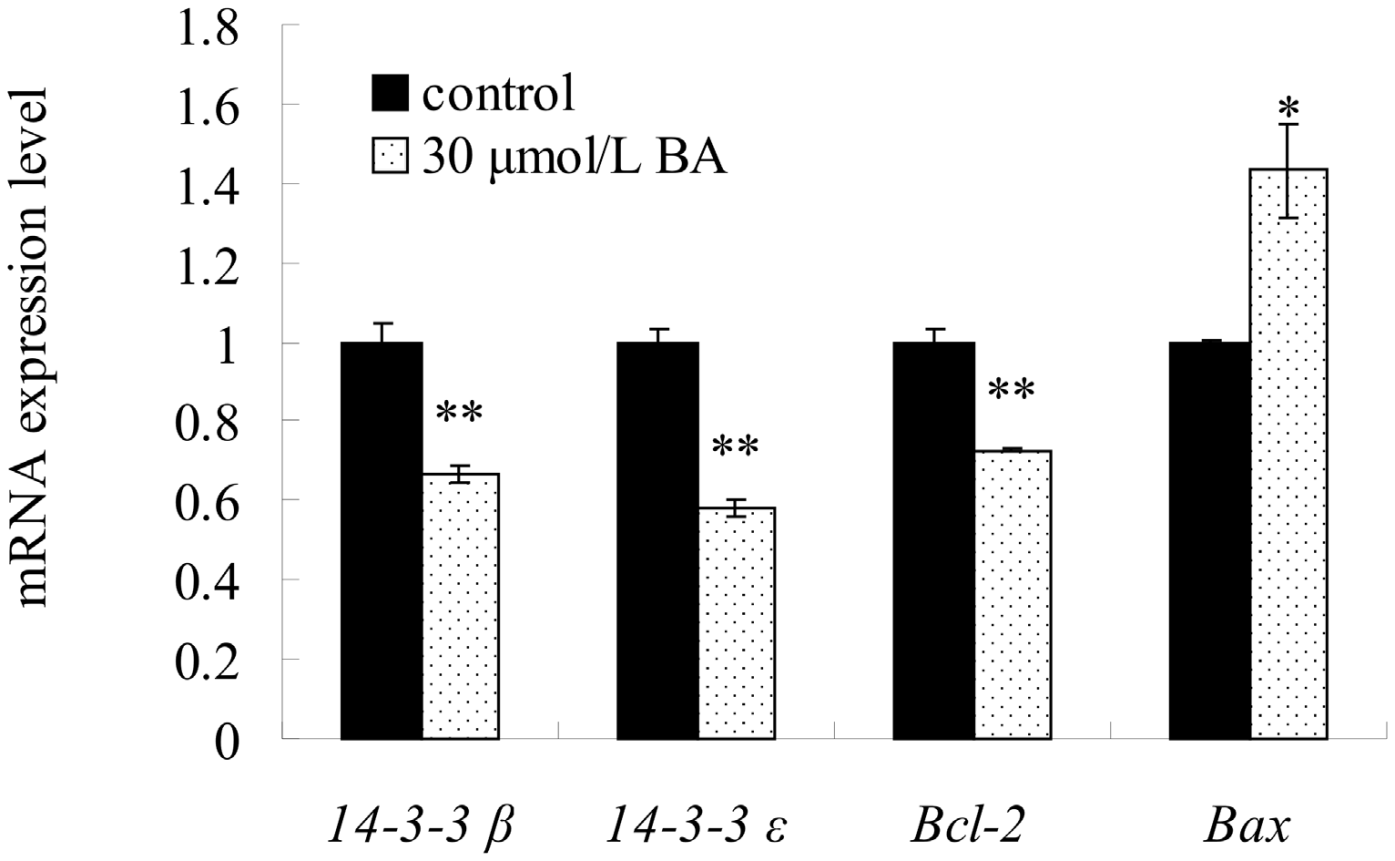
The mRNA expression of *14-3-3β*, *14-3-3ε*, *Bcl-2*, *Bax* were detected by qRT-PCR. HeLa cells were treated with 30 µmol/L BA for 24 h and then Total RNA was isolated using a Trizol reagent. Columns show mean values of three experiments (±SD). *p<0.05, **p<0.01 compared with the control group (0 µmol/L).

Because 14-3-3 proteins are involved in many physiological pathways, including growth and apoptosis, expression changes in the two genes that encode the identified 14-3-3 family of proteins were selected for further analysis by qRT-PCR. The down-regulation of the two selected genes (*14-3-3β* and *14-3-3ε*) at the transcription level was consistent with the 2-DE data. The expression levels of *14-3-3β* and *14-3-3ε* were significantly down-regulated by BA treatment. Genes *Bcl-2* and *Bax* were investigated to further explore the roles of ROS accumulation in BA-induced apoptosis because the mitochondrial pathway is regulated by prosurvival Bcl-2 and proapoptotic Bax. The expression level of *Bcl-2* was observed to be significantly lower than the control level. In contrast, the expression of proapoptotic *Bax* was significantly increased compared to the controls by qRT-PCR (Fig. 6).

## Discussion

BA, a naturally occurring pentacyclic triterpenoid with the potential to kill melanoma cells [1], has been shown to induce apoptosis in many cancer cell lines [2]–[8]. BA has been reported to be devoid of cytotoxic effects against healthy cells. Normal cells, such as dermal fibroblasts, peripheral blood lymphoblasts [2], melanocytes [14] and human astrocytes [15], have been shown to be resistant to BA treatment in vitro. Several studies have provided considerable insight into BA-induced cytotoxicity. BA also suppressed tumor growth in vivo in a number of animal studies. In a xenograft mouse model of ovarian cancer administration of BA significantly increased the survival time [25]. Schettino, M T took advantage of BA in treatment of Human Papilloma Virus which is considered necessary for the development of cervical cancer, the results suggest BA may be one of promising drugs to treat cervical cancer [26]. The aim of the present study was to elucidate the molecular mechanism(s) of the apoptotic effects of BA in a cervical cancer cell line (HeLa) and to investigate the effects of BA on the growth of HeLa cells.

To determine the appropriate concentration of BA for the proteomic experiments, HeLa cells were treated with a series of BA concentrations for 24 h, 48 h and 72 h, and then cell viability was measured by a MTT assay and the apoptosis rate was analyzed by flow cytometry. The result revealed the BA inhibits the proliferation of HeLa cells in a dose- and time-dependent manner, and the IC_50_ was 25.93±1.87 µM for 72 h, which was consistent with the IC_50_ (26.0±2.1 µM) from Rita C tested HeLa cells exposed to BA treatment for 72 h [24]. Significant growth inhibition and cell apoptosis were observed when the cells were exposed to 30 µmol/L BA for 48 h. To comply with a standard that cell apoptosis should be induced but enough living cells should also be maintained for protein extraction and proteomic study, 30 µmol/L BA was chosen for treatment of the HeLa cells.

Proteomics offers a method for identifying which proteins mediate apoptotic pathways in cells treated with chemotherapeutic agents [27]–[29]. Because the molecular mechanism involved in the induction of proliferation inhibition and apoptosis by BA is still not clear, we implemented a proteomics scheme to globally search for differentially expressed proteins in HeLa cells affected by BA. Most proteins (30 spots) were decreased and few proteins (6 spots) were enhanced by BA treatment. Functional category analysis suggests that the alterations in protein expression after BA treatment are associated with different biological processes and molecular functions. These results suggest that BA-induced apoptosis of HeLa cells mainly relies on inhibition of proteins involved in synthesis and metabolism.

Among the fifteen metabolic process related proteins, two of the up-regulated proteins (HSPA5, spot 613 and PLIN3, spot 248) are involved in the endoplasmic reticulum (ER) pathway, which is involved in complex apoptotic responses. The ER stress response can promote cellular repair and sustained survival by reducing the load of unfolded proteins through global attenuation of protein synthesis or up-regulation of chaperones, enzymes, and structural components of the ER [30]. Although the mechanism is still unclear, when ER stress is overwhelming, cells undergo apoptosis [31]. The glucose-regulated protein HSPA5 (spot 613), which is an ER-resident chaperon, responds to the ER pathway through a network of regulators and novel mechanisms leading to growth arrest [32]. Cargo, including PLIN3 (spot 248), is translocated into the ER and is then incorporated into small vesicular carriers that mediate transport to Golgi compartments [33]. Another up-regulated protein is IL18 (spot 27), a novel pro-inflammatory cytokine that induces expression of tumor necrosis factor a (TNF-a) and Fas ligand, as well as nuclear translocation of nuclear factor kB (NF-κB) [34], [35]. NF-κB is a key regulator of stress-induced transcriptional activation, and BA has also been reported to inhibit inflammatory activation of NF-κB [36]. In the present work, up-regulated protein expression was detected, which may mediate the ER process of BA in HeLa cells.

Two down-regulated antioxidant activity proteins, UGDH (spot 526) and PGD (spot 1120), catalyze the oxidation of C6 alcohols into carboxylic acids in two successive NAD^+^-dependent steps without the release of an intermediate aldehyde, and these proteins are very important for the electron transfer chain in mitochondria [37], [38]. Additionally, the antioxidant activity protein PRDX6 (spot 542) may provide protection from nitroxidative stress and repair oxidatively damaged molecules, and this protein plays an important role in scavenging ROS in physiological conditions [39]. At the same time, the level of ROS production detected by flow cytometry was increased after the treatment of HeLa cells with BA, and this result is in accordance with the results of previous oxidoreductase activity assays and may help to explain the apoptotic effects of BA. These results indicate that the mitochondria pathway was activated by BA, which then induced apoptosis of the HeLa cells. Oxidative stress has been known to be a mediator of apoptosis induced by a variety of triggers, and the mitochondria are regarded as sensors of oxidative damage and may play a major role in apoptosis [40], [41]. The Bcl-2 family of proteins can regulate the mitochondria pathway through altering mitochondrial membrane permeabilization [42]. Antiapoptotic Bcl-2 and proapoptotic Bax are very important for mediating the outer mitochondrial membrane permeabilization and mitochondrial pathway apoptosis [43]. We found that BA treatment suppressed the expression of *Bcl-2* transcripts, facilitated the expression of *Bax* by qRT-PCR. This provides further indication that the mitochondria participate in the intrinsic apoptosis pathway involved in the BA-induced apoptosis mechanism.

Based on the STRING analysis, a connected network showing a reliable relationship among the response proteins associated directly or indirectly with apoptosis was constructed. Moreover, a KEGG tool was applied to further investigate the underlying pathway. Four key networks were identified to be involved in a variety of signaling and metabolic pathways. The top signaling pathway was the “neurotrophin signaling pathway”. In the present study, down-regulation of two cellular proteins (14-3-3β and 14-3-3ε) involved in the neurotrophin signaling pathway were validated by qRT-PCR analysis. These observations are consistent with the previous results of the proteomic analysis. It has been reported that 14-3-3 proteins play an important role in the regulation of a wide variety biological processes, including proliferation, cell cycle and apoptotic signaling [44]. The pro-apoptotic family member Bad protein is one of the targets of 14-3-3 proteins. It has been reported that the apoptotic mechanism of drugs may be induced by the dissociation of Bad from 14-3-3β via the translocation of Bad to the mitochondria in retinal ganglion cells [45]. These findings suggest that Bad dissociation from 14-3-3 is a key mediator in BA-induced apoptosis through the disruption of mitochondrial membrane potential. 14-3-3ε isoforms identified in cancer have been reported to bind to transcriptional coactivators [46], [47]. These data suggest that down-regulation of 14-3-3 could provide useful information regarding therapeutic targets of cervical cancer by BA. Taken together, these results may expand our understanding of the anticancer mechanism of BA on HeLa cells from a protein and molecular perspective.

In summary, BA showed a strong inhibitory effect on HeLa cell growth at pharmacological concentrations (30 µmol/mL), and 36 differentially expressed proteins were identified in HeLa cells after exposure to BA using both 2-DE and MADLI-TOF analysis. Proteomic studies confirmed that BA-induced apoptosis involved molecular alterations in ROS generation, 14-3-3 protein regulation and related regulatory proteins in HeLa cells. In particular, almost all the proteins involved biological processes and molecular functions were down-regulated in BA-treated HeLa cells compared to control cells. The findings of this study suggest that BA inhibits HeLa cell growth, alters apoptosis related proteins and induces apoptosis not only via the ROS-medicated mitochondria pathway but also triggers the ER pathway, and these insights may be helpful to understand the molecular mechanism of BA's anti-tumor effect in HeLa cells. Further study on the pathway by which BA induces apoptosis would illuminate the molecular mechanism of mitochondria induced apoptosis.

## Supporting Information

Figure S1 **BA-induced apoptosis in Hela cells using annexinV-FITC/PI.** (A) Cells were treated with different concentrations (30 µmol/L, 50 µmol/L) of BA for 48 h. (B) Flow cytometric histograms. Columns show mean values of three experiments (±SD). **p<0.01 compared with the control group (0 µmol/L).
(TIF)

Table S1 **Peptide sequences and observed m/z of the identified proteins in MALDI-TOF/TOF mass spectrometry analysis.**
(XLS)
