# Supplementary figures and images for: Proteomic Investigation into Betulinic Acid-Induced Apoptosis of Human Cervical Cancer HeLa Cells
Source: PLoS One. 2014 Aug 22;9(8):e105768. doi: 10.1371/journal.pone.0105768 (PMC4141803; doi:10.1371/journal.pone.0105768)

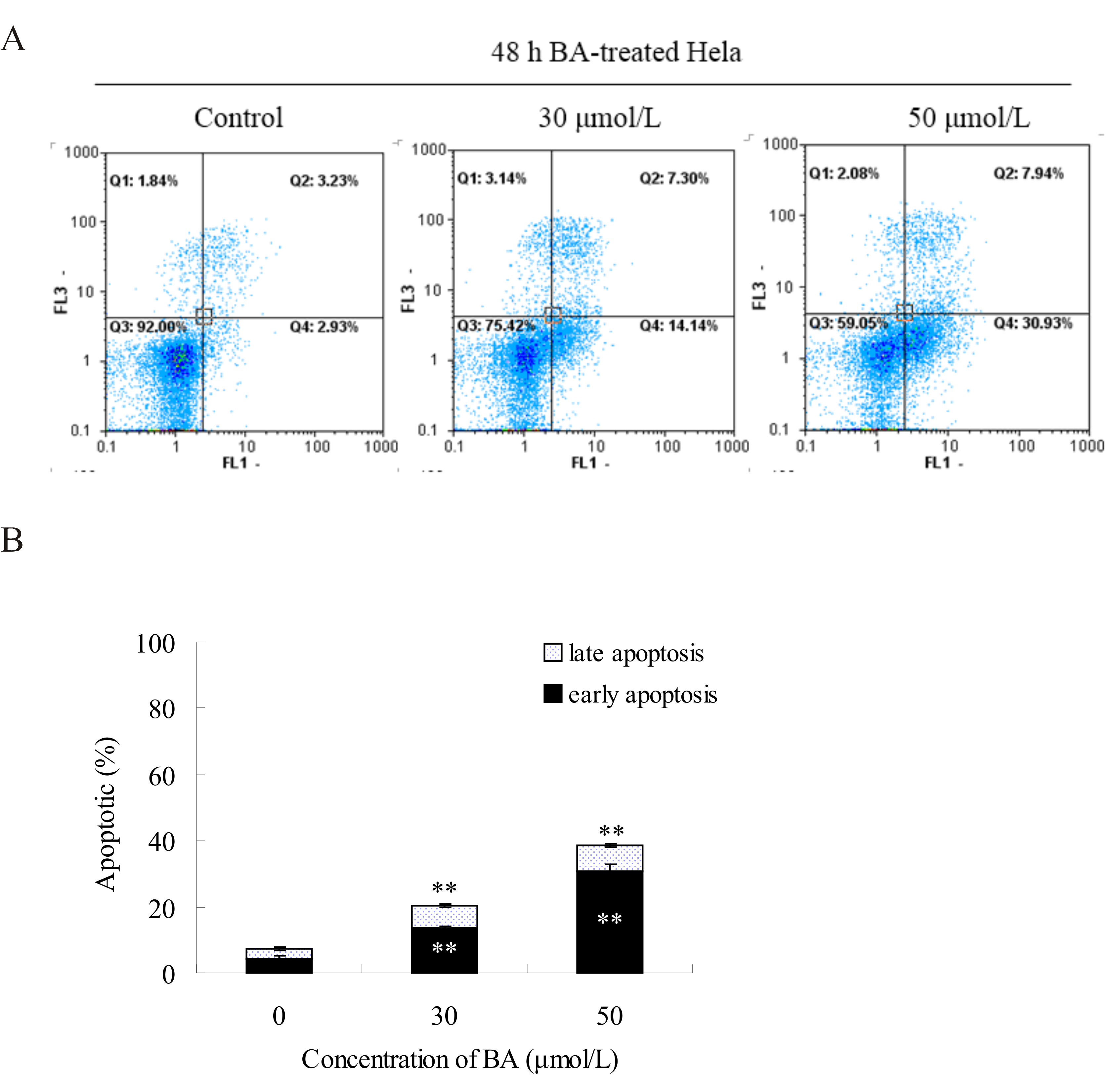

Supplement: Figure S1 — BA-induced apoptosis in Hela cells using annexinV-FITC/PI. (A) Cells were treated with different concentrations (30 µmol/L, 50 µmol/L) of BA for 48 h. (B) Flow cytometric histograms. Columns show mean values of three experiments (±SD). **p<0.01 compared with the control group (0 µmol/L). (TIF) [file pone.0105768.s001.tif]
